# Sensor Buoy System for Monitoring Renewable Marine Energy Resources

**DOI:** 10.3390/s18040945

**Published:** 2018-03-22

**Authors:** Emilio García, Eduardo Quiles, Antonio Correcher, Francisco Morant

**Affiliations:** Instituto de Automática e Informática Industrial, Universitat Politècnica de València, 46022 Valencia, Spain; egarciam@isa.upv.es (E.G.); ancorsal@ai2.upv.es (A.C.); fmorant@isa.upv.es (F.M.)

**Keywords:** marine sensor system, sensor buoy, remote monitoring, marine energy, renewable energy

## Abstract

In this paper we present a multi-sensor floating system designed to monitor marine energy parameters, in order to sample wind, wave, and marine current energy resources. For this purpose, a set of dedicated sensors to measure the height and period of the waves, wind, and marine current intensity and direction have been selected and installed in the system. The floating device incorporates wind and marine current turbines for renewable energy self-consumption and to carry out complementary studies on the stability of such a system. The feasibility, safety, sensor communications, and buoy stability of the floating device have been successfully checked in real operating conditions.

## 1. Introduction

The objective of the EU Renewable Energy Directive is to ensure that by 2020, 20% of the energy consumed comes from renewable sources. Among the best candidates that are experiencing development are marine renewable energy sources such as wind energy, wave energy, and energy extracted from marine currents.

Since over 25 years ago, the convenience of moving wind generation parks to the marine environment has been considered [1] in order to achieve more favorable conditions for generation, with higher intensity wind flows, on average 30% higher than those achieved on the mainland. At the same time, offshore windfarms reduce the inconveniences that onshore windfarms have, regarding aviary fauna and visual and noise pollution. Additionally, operating conditions in open sea allow the installation of large-scale wind turbines (WTs), with rotor diameters that already exceed 120 m and are therefore capable of generating large amounts of power due to the area they are able to cover.

Marine energy generation has been pushed up recently with the installation of diverse systems for the use of waves and marine current energy. Specifically, marine current turbine (MCT) technology is being seriously considered among research groups and companies. Projects like the SeaFlow (300 KW) at Lynmouth on the North Devon Coast or the more recent SeaGen (1.2 MW) in Strangford Narrows, Northern Ireland, prove the feasibility of this type of generation [2].

Both onshore (shoreline and nearshore) and offshore wave power generators are experiencing great development with new and diverse operational systems that have not yet reached their maximum potential [3,4]. This type of wave converters are based on oscillating water columns (OWC) [5], sea-wave slot-cone generators (SSG) [6], overtopping flow devices (OTD) [7,8,9], point absorbers (PA) [10], oscillating wave surge converters [11], attenuators [12,13], submerged pressure differential devices [14], bulge wave [15], and rotating mass [16] technology. 

According to the Intergovernmental Panel on Climate Change (IPCC) estimation, considering all areas with energy density from waves greater than 5 kW/m, there is a potential of around 32,000 TWh per year [17]. Specifically, for Europe, a potential of 1000 TWh/year is estimated [18].

As a prerequisite for the development of such projects, it is necessary to carry out preliminary economic feasibility studies that analyze the amount of energy resources existing in the sites under consideration. In these studies, time series of data from historic years are used [17]. In onshore wind generation parks, it is usual to carry out studies with a typical duration of two years.

For this reason we have designed and developed a sensor floating system able for long-period observation of energy resources in the marine environment. In the context of the development of our objectives, a comparative study of the state of the art floating devices dedicated to the measurement of marine environment parameters was made. 

The Seawatch buoy [19] is a floating platform capable of housing a plethora of sensors whose purpose is to measure atmospheric and meteorological parameters. Additionally, the buoy has a directional wave sensor. The Wavescan buoy [20] is a lenticular buoy with an approximate diameter of 2.75 m weighing about 1000 kg. It includes a sensor for directional wave measurement and an upper structure to support meteorological sensors. Wavec buoys and Waverider buoys [21] allow measurement of scalar parameters and directional parameters. The Motus Wave buoy has complete sensor equipment with the capacity to measure a wide range of marine parameters [22]. Metbuoy offers complete instrumentation for measurement of meteorological parameters [23]. The EBM-OC Buoy [24] is sold with a basic configuration, which is expandable upon customer demand. Mobilis [25] is specialized in offering customized buoys with meteorological measurement and marine parameter technology.

In Ref. [26] a low-cost prototype sensor with a microcontroller and digital sensors for measuring temperature, light, and pressure, housed in a miniaturized buoy, has been developed for sampling sea surface temperature, light intensity, and sea level changes. In Ref. [27] a low-cost buoy system was developed to perform remote monitoring of atmospheric temperature and marine pressure and temperature. These low cost approaches are not suitable for long observation periods under adverse weather conditions. The power supply of the cases studied above was based on the use of solar panels. In Ref. [28], a Global Navigation Satellite System (GNSS) buoy that utilizes a Virtual Base Station (VBS) combined with the Real-Time Kinematic (RTK) positioning technology was developed to monitor water surface elevations in estuaries and coastal areas.

None of the existing solutions were well suited to the particular objectives of our research project. The main focus of our study is the search for marine energy resources and these requirements have been included in the buoy design. In this paper, we present a multi-sensor floating prototype for assessing marine renewable energy resources.

The main objectives of our project are:

(a) To design a multi-sensor buoy to monitor offshore sites with suitable wind, wave, and marine current resources for power generation in the marine environment.
(b) To check the feasibility, safety, and autonomous operation of the designed platform for remote data acquisition.
(c) To design a test bench for the control and diagnosis of marine energy generators.

In Section 2 of this work, the structural design and some hydrodynamic and hydrostatic results of simulations using OrcaFlex (Version 9, Orcina Ltd., Cumbria, UK) and NX software (Version 9, Siemens PLM Software, Plano, TX, USA) are shown. Section 2 also presents electrical and electronic components of the system, including sensors and transmitter/receiver data communication systems. In Section 3, some test results are presented regarding MCT re-design and sensor data transmission. In Section 4, the results are discussed. Finally, in Section 5, conclusions about our work can be found.

## 2. Sensor Buoy System Design

We have implemented a multifunctional floating system for monitoring renewable marine energy resources. This system integrates offshore renewable electricity generation from both wind and sea currents to charge the batteries. In this way, the system is provided with energy for self-consumption. It can also be used as a test bench for stability control, condition monitoring, fault diagnosis, and predictive maintenance of renewable offshore power generation systems.

In the design phase of the project we have developed the following tasks:

- Mechanical, hydrostatic, and hydrodynamic design.
- Utilization of various modeling and simulation techniques for designing and tuning the prototype.
- Selection of the appropriate sensors and energy generators.
- Selection and configuration of the communication systems and radio devices.

### 2.1. Mechanical Design

**Figure 1:**
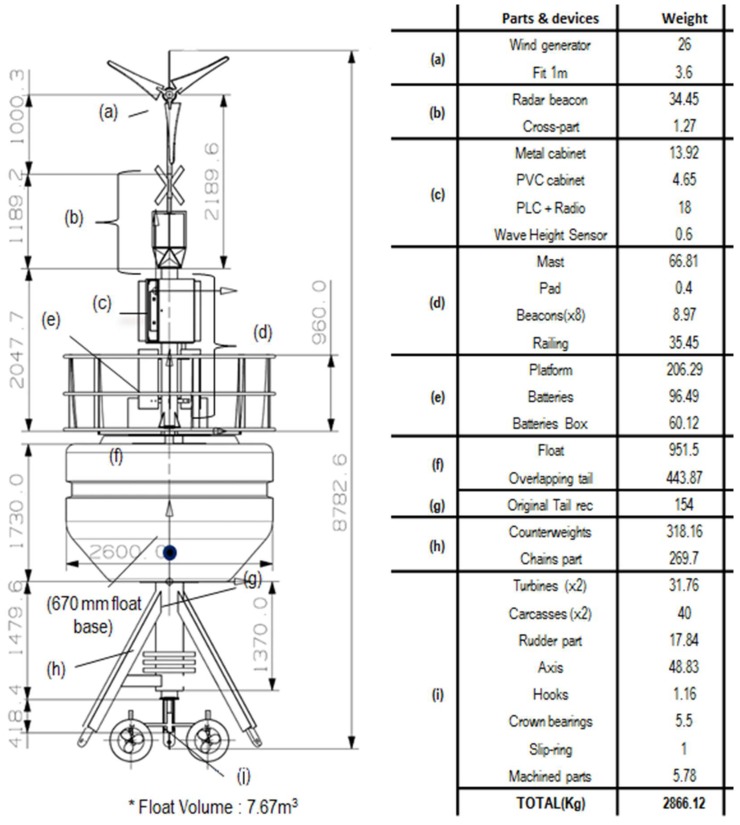
Buoy dimensions (m) and weights (kg).

The hydrostatic-hydrodynamic design of the system was carried out mainly by Orcina OrcaFlex software [29]. OrcaFlex is a software package for static and dynamic analysis of offshore marine systems. Among other capabilities, it allows the simulation of 6 degrees of freedom systems with a full 3D representation. Specifically, this software allows one to carry out simulations of the catenary anchor system incorporating environmental conditions as currents, wind, waves, eddies, and so on, checking the stability and buoyancy of the prototype and the stresses in each of the mooring cables, and selection of materials, types, and weights able to support the catenary tension. This software has been used to simulate the complete system behavior under average and extreme weather conditions (currents, waves and wind). We have successfully managed to perform the modeling and simulation of the full buoy system including the catenary system and the turbine rotating system. Figure 1 shows the buoy dimensions and weights.

This modelling software in parallel with Unigraphics NX [30] helped us to obtain mass centers and moments of the buoy, since these parameters were changing during the design process as elements were added. All the elements of the buoy are faithfully represented in Unigraphics NX (sizes, materials, densities, and weights). This software was also used to study the increase in the speed of water flow through the rotor of MCT internal nozzles [31]. Combined use of the modeling and simulation software (Unigraphics NX and Orcina Orcaflex) has served for parts redesign and feedback of several of the modifications made to the buoy.

Basically, to model a floating system of 6° of freedom in Orcina Orcaflex, the base structure is defined by superimposed cylinders. Next, the hydrostatic properties of each of the cylinders are added, and the center of mass, weight, and inertia moments of the assembly are specified. 

A MCT turning system with a rudder was designed. This design allows the passage of both the power generated in the marine turbines and the data coming from the underwater line current sensors through the use of a rotating electrical connector (slip ring). The rotating axis for the MCT and rudder system was also modeled with Orcina Orcaflex. Lift and drag coefficients corresponding to a NACA 63-012 rudder were introduced in the model to implement the rudder of dimensions 40 × 70 cm. 

**Figure 2:**
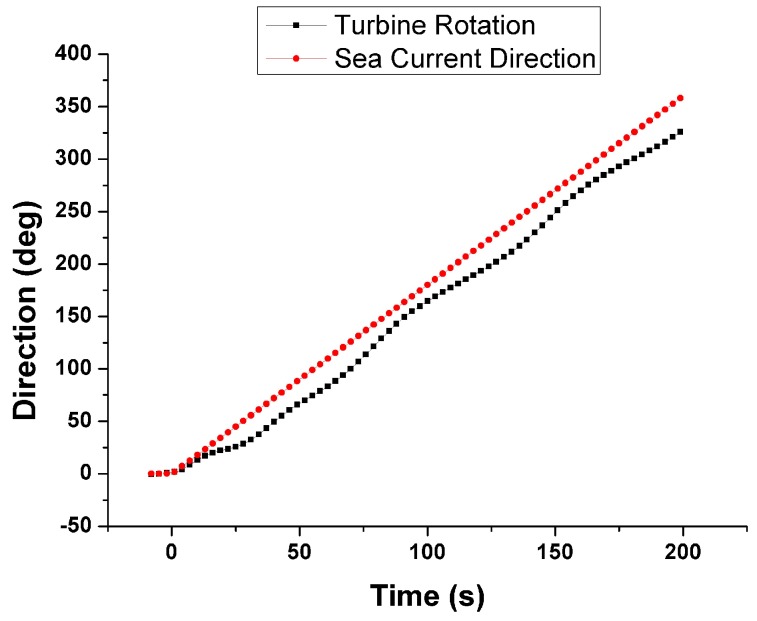
Following of the MCT-rudder system to variations of the sea current.

With this setup, the full buoy body and the inner rotating system for the turbines were successfully modeled. In Figure 2, we can see how the group of the two marine current turbines and the rudder follows the direction of the environment marine current (0.2 m/s) when varying it from 0° to 360°. No wind and no waves were simulated.

In order to see the full response of the buoy to hard environmental conditions, a full simulation was done with the following set up: 0.5 m/s marine current speed at 0°, 10 m/s wind speed at 180°, 3 m wave height, and 5 s wave period at 0° direction.

**Figure 3:**
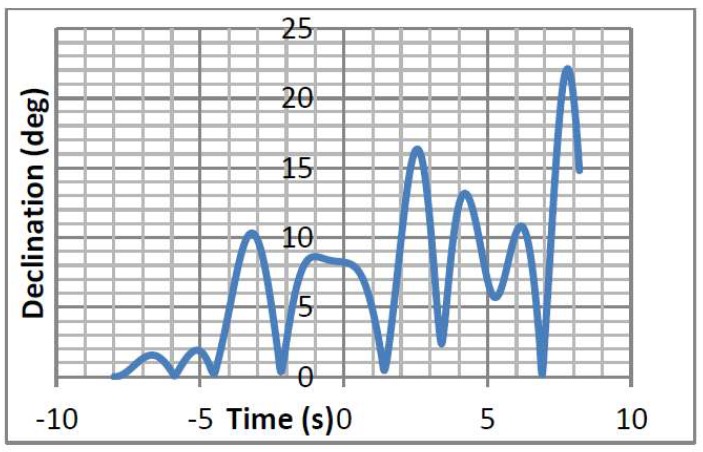
Buoy declination under adverse weather conditions: Currents 0.5 m/s, wind 10 m/s, wave height 3 m/5 s period.

**Figure 4:**
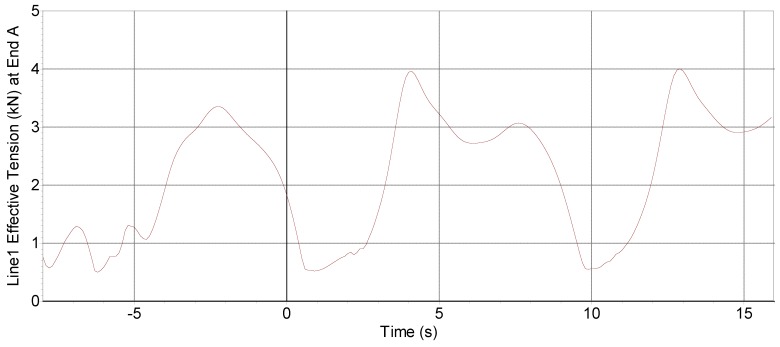
Effective catenary strain under adverse weather conditions: Currents 0.5 m/s, wind 10 m/s, wave height 3 m/5 s period.

**Figure 5:**
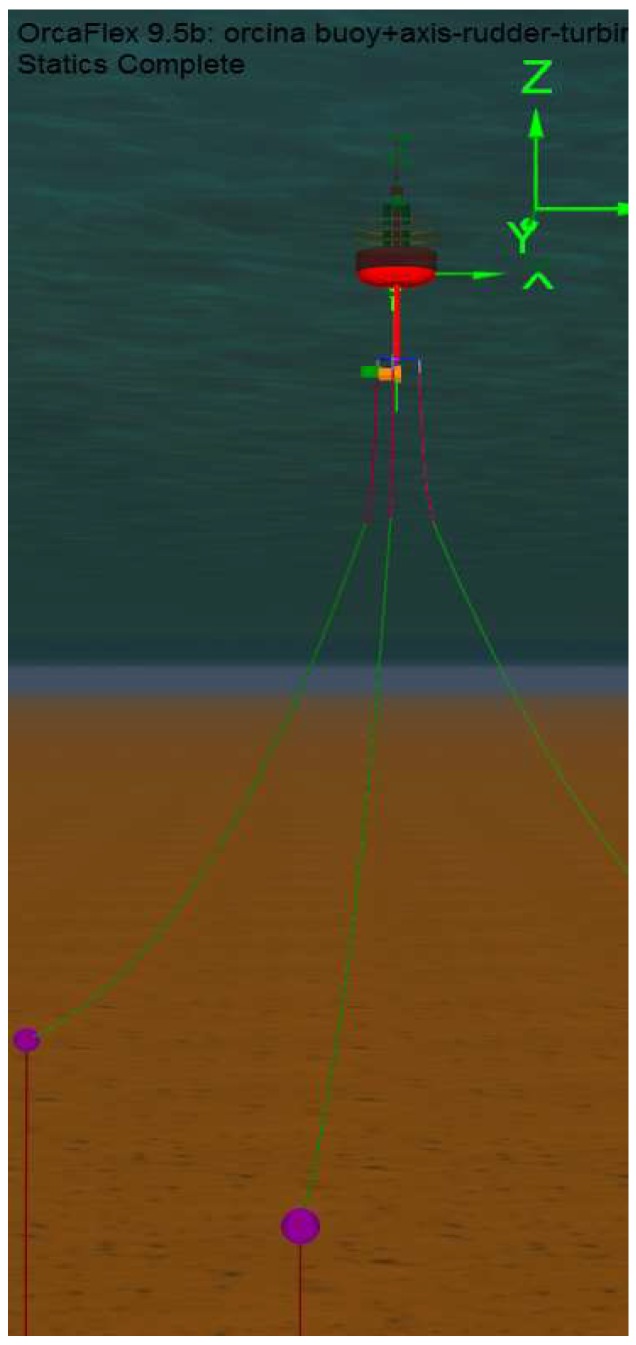
Orcina Orcaflex model prototype with catenary anchoring and intermediate floats.

With these conditions, Figure 3 shows the declination of the buoy and Figure 4 shows the tension supported in one of the lines of the catenary system. This information served for the design in Orcina Orcaflex of the catenary system, as shown in Figure 5. In order to lighten the buoy’s total load, hybrid chains of the metal-polyester-nylon-metal type were used. Table 1 shows the composition length and weights of each of the sections. The stresses that support these chains are much greater than the maximum tension that would occur in adverse sea conditions. The study was carried out by simulation with Orcina Orcaflex software [29]. The use of intermediate floats of 0.5 m^3^ serves to create a virtual bottom at 30 m depth giving more stability to the buoy and lightening its load. The system of triple catenary joins a triangular metal piece whose arms surpass the turbines to avoid entanglement.

### 2.2. Electronic Design

The system is equipped with three subsystems:

(a) Sensors Subsystem, including a height wave sensor [32], two current meters [32], and a PB200 weather-station [33], that integrates several functionalities to be detailed in the next subsection.
(b) Energy generation and storage subsystem, incorporating a Wind Turbine (WT) [34], two Marine Current Turbines (MCTs) [34] and a small photovoltaic panel in order to ensure a redundant source of power in situations of long periods of atmospheric calm. The energy storage subsystem is formed by an A00 RG VW-50-24 and two A00 RG S1B-24 from Ampair (Dorset, UK), and a Tristar 45 charge controller from Morningstar Corporation (Newtown, PA, USA), and two Absorbed Glass Mat type AGM batteries from VETUS (Schiedam, The Netherlands) [35].
(c) Data acquisition, control, and radio communication subsystem, formed by an ACE3600 PLC [36].

**Figure 6:**
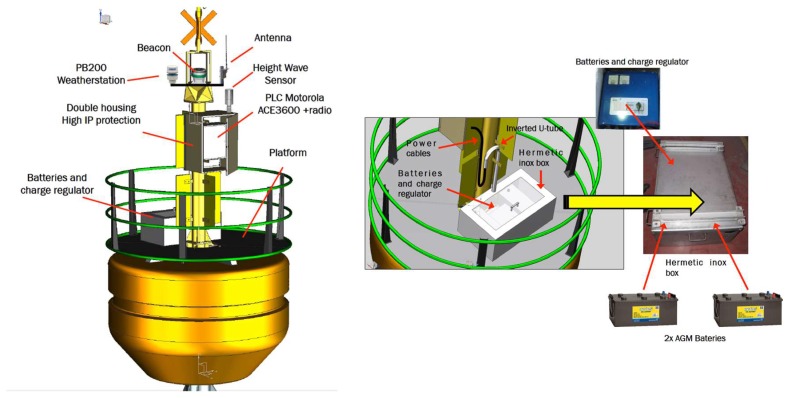
Devices location on the mast and platform buoy.

Figure 6 shows the location of most sensors and electronic devices, as well as the energy storage subsystem on the mast and platform buoy, respectively.

#### 2.2.1. Sensor System Components

The main objective of this multifunctional system is to make observations and measurements of energy resources based on wave, current, and wind energy in the marine environment. Next, the characteristics of the selected sensors are detailed.

##### Wave Height Sensor

The energy coming from waves is basically derived from the sum of their potential and kinetic energy. For regular waves, the expression of its power is given by

(1) $$P=981.21H^{2}T\;\left[W/m\right]$$

As shown in expression (1), the power to be generated per wave front meter depends on the parameters of wave height *H* and its period *T*.

**Figure 7:**
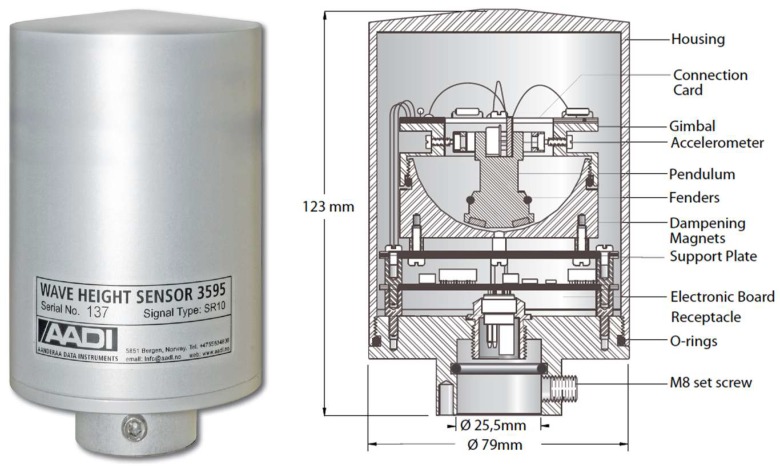
Wave height sensor.

Therefore, in order to carry out a preliminary evaluation of the existing wave energy resources, the wave height sensor 3595 from Aanderaa Data Instruments was selected (Figure 7). The outputs from the sensor are significant wave height *Hs* and significant wave period *Ts*.

The sensor is mounted on the floating system that will follow the movement of the waves. The accelerometer mounted on the pendulum senses the movement of the buoy.

The accelerometer’s sensitive axis is kept vertical, plus or minus the pendulum’s displacement angle. Since the accelerometer also senses gravity, the variation of the maximum acceleration measured during the pendulum’s oscillation period is a measure of the vertical acceleration caused by the waves.

The acceleration is sampled four times a second and then integrated twice to give the vertical distance the accelerometer has traveled. This is the distance from the top to the bottom of a wave.

The output from the sensor is the significant wave height which is the average height of the upper third of all waves during the measuring interval and wave period [32].

It has its own output in the so-called SR10 format (digital signal). There are two options to process this signal, either acquire the manufacturer’s “datalogger” or interpret the signal. The second option was chosen using a PIC microcontroller together with specific circuitry. The adaptation circuit board converts the serial digital signal of the wave height sensor (SR10) into an analog voltage in the acquisition range of the Motorola ACE3850. The timing diagram of this communication format is described Appendix A.

##### Marine Current Sensor

Marine Current Turbines (MCTs) and the Wind Generators (WTs) share the same physical principle that allows energy generation. It is about taking advantage of the kinetic energy resulting from the wind and marine currents. MCTs have the advantage that the density of the sea water is approximately 800 times greater than that of air, which establishes favorable generation conditions for them, taking into account the density parameter in Equation (2) [2].

*P =* 1/2*ρ_w_CpAV*^3^ (2)

where *P* is the generated power, *V* the current speed, *A* the rotor area, and *ρ_w_* the water density. As stated in Ref. [2], *Cp* is known as the power coefficient and is the percentage of power that can be extracted from the fluid stream and takes into account losses due to the Betz law and those assigned to the internal mechanisms within the converter or turbine. For wind generators, *Cp* has typical values in the range 0.25–0.3. The upper limit is for highly efficient machines with low mechanical losses. For marine turbines, *Cp* is estimated to be in the range 0.35–0.5 [37]. Other specific characteristics of the MCTs have to do with the loads and ranges of flows to be supported, as well as the effects of cavitation [2]. 

Consequently, current sensors will be used to evaluate the existing energy resources associated with marine currents. In our design, the buoy is equipped with two marine current meters located at two different depths, one is mounted near the surface, and the other one is close to the bottom, indicating current intensity and heading. The objective is to get information on surface currents versus bottom currents, which are usually colder and denser.

**Figure 8:**
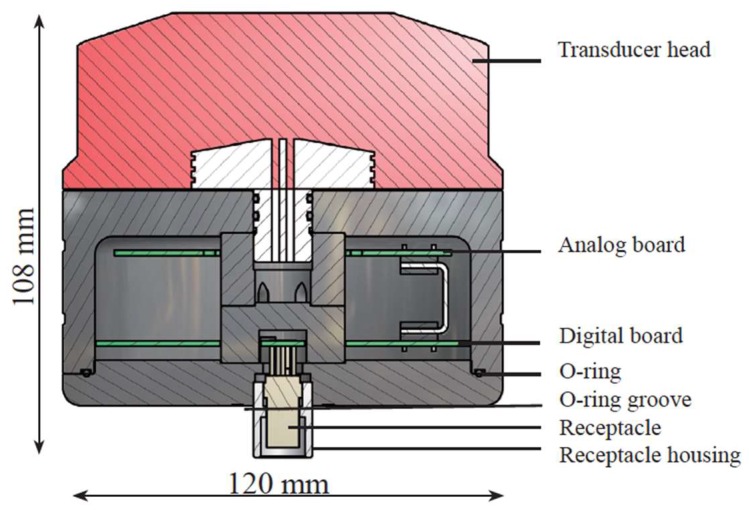
Marine current meter sensor.

These current sensors are two doppler current sensors mounted at 4 m and 58 m deep, respectively. The model is the ZPulse DCS 4420 of Aanderaa data Instruments (Figure 8). It uses a RS232/RS422 communication protocol and measures current direction and intensity up to 300 m depth.

The sensors are based on the backscatter acoustic Doppler principle using ZPulse™ technology. Complex acoustic pulses comprising several distinct frequencies are combined into a single acoustic pulse that is sent out at right angles at regular intervals.

The ZPulse™-based Doppler current sensor separates the received signal into different frequency bands, one for each frequency in the transmitted signal. Further, it analyses the frequency shift using a high-speed DSP. An ARMA based parametric model processing algorithm is used to find the Doppler frequency. The ZPulse™ technology reduces statistical variance. This again reduces the required number of pings needed in order to achieve an acceptable statistical error.

The measured current speed vector is corrected for the sensor’s heading and tilt. This is done by using a built-in electronic compass and a tilt sensor to obtain a true North and East current speed reading.

The solid-state sensor is well suited for monitoring low current speeds due to having no moving parts. Because the sensor starts measuring in an area 0.4 m to 1.0 m from the instrument, the effect of marine fouling and local turbulence is minimized.

Marine currents are influenced by many causes, but on a global scale it is 3-dimensional, with surface movements where the wind is the main thrust force, and with vertical movements affected by salinity and temperature. Superficial currents give way to deep currents of different characteristics, by convective movements, deflection, and friction effects [38,39,40]. 

**Figure 9:**
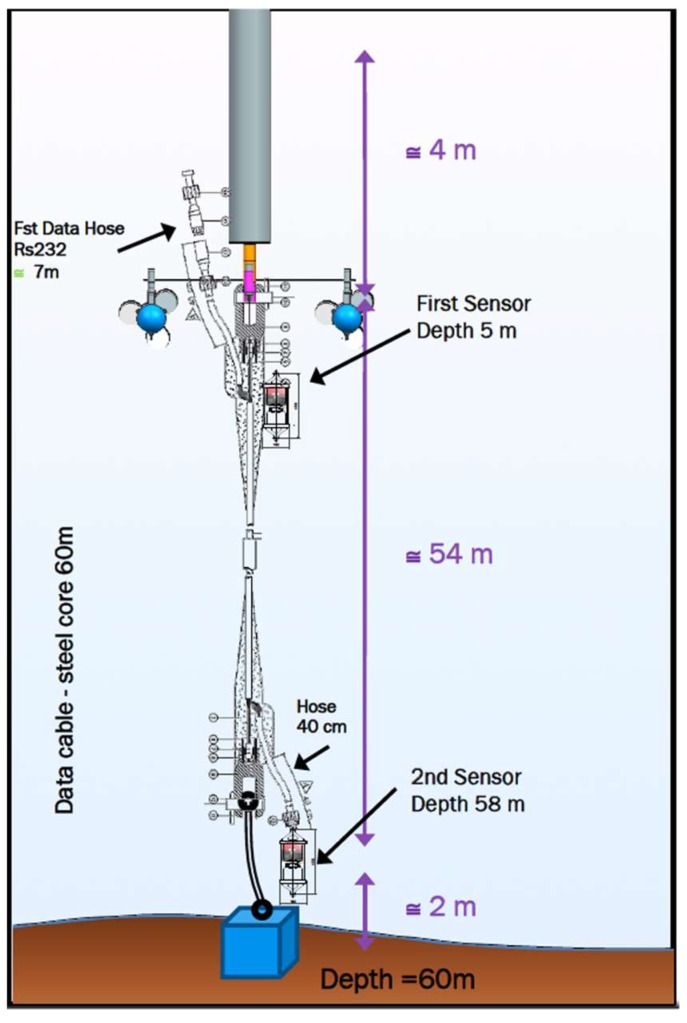
Current meter sensor locations depth.

It was decided to make observations on both surface and bottom sea currents due to the lack of previous local studies, unknown models, and seasonal changes affecting the currents. Figure 9 shows the current meter sensor locations.

##### Anemometer & Weather-Station Instrument

As with marine currents, the primary source of the energy resource to be considered susceptible to measurement is the kinetic energy due to wind speed. The fraction of power extracted from the wind by a wind turbine is given by the parameter *Cp*, which is known as the efficiency coefficient, in such a way that the power generated is given by the expression:

(3) $$P=Cp\;\rho \;r^{2}v^{3}$$

where *P* is the generated power, *v* wind speed, *r* rotor radius, and *ρ* air density. The efficiency coefficient is not constant, but varies with the wind speed *v*, the rotational speed of the turbine, and the parameters of the blade such as the angle of attack *θ* and the pitch angle of the blade. When the rotational speed varies, it causes the angle of attack to change as well. 

**Figure 10:**
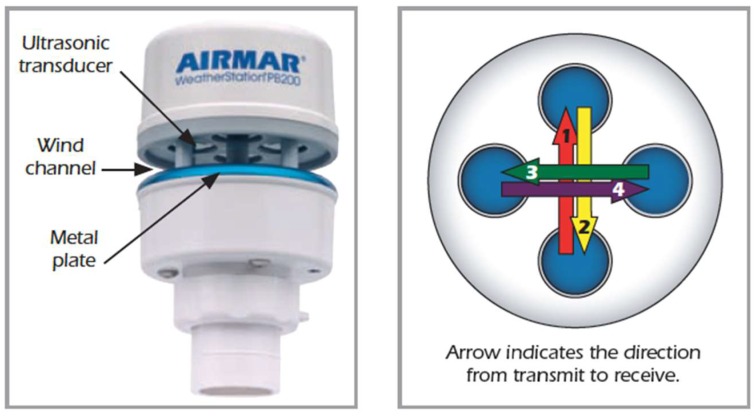
The multisensor PB200 weather-station.

For the measurement of energy resources associated with the wind, an ultrasonic weather-station instrument of Airmar Technology Corporation (Milford, NH, USA) has been included [33] (Figure 10).

The PB200 Weather-Station^®^ instrument [33] is designed to output time series of data of instantaneous changes in the weather. Wind speed and direction are measured using four ultrasonic transducers as an anemometer without moving parts. This configuration results in better durability and reliability. The internal WAAS/EGNOS GPS engine and three-axis, solid-state compass make it possible for the PB200 to provide both apparent and true wind speed and direction without the need to add additional sensors. The WAAS/EGNOS GPS provides navigation data as well as magnetic variation and is suitable as a primary GPS source, so it helps with positioning and location information, which is very helpful in the case of recovery or maintenance tasks. Additionally, it is equipped with internal temperature and barometric pressure sensors that help to perform trending analysis and forecasts on changing weather patterns. 

The ultrasonic anemometer measures apparent wind speed and direction using four ultrasonic transducers, visible through the four holes in the top of the sensor’s wind channel. These transducers operate in pairs. One transducer injects a pulse into the air, the pulse bounces off the metal plate at the bottom of the wind channel and is carried by the wind to arrive at the listening transducer a short time later. When there is no wind, the pulse travels at the speed of sound from the sender to the receiver. Whenever the wind is blowing in that direction, the pulse will arrive sooner than if the air is still. Similarly, whenever the wind is blowing in the opposite direction, the pulse will arrive later than if the air is still. The four transducers take turns in sending and receiving pulses.

A microprocessor within the weather-station instrument then combines the measurements from all four transducers to calculate the resultant wind speed and direction. Throughout this process, the sensor monitors the air temperature to compensate for the fact that the speed of sound in air changes with temperature. The PB200 can communicate using both NMEA183 (RS232) or NMEA2000 (a CAN based Protocol).

#### 2.2.2. Power Sources System

**Figure 11:**
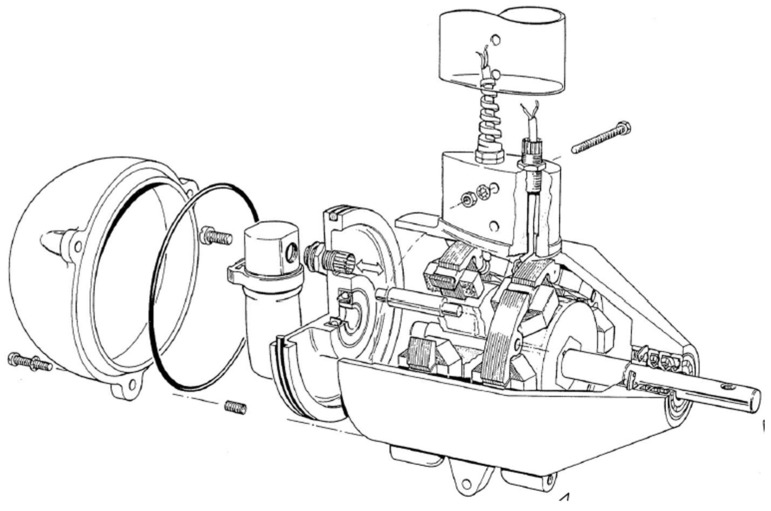
Current Turbine.

**Figure 12:**
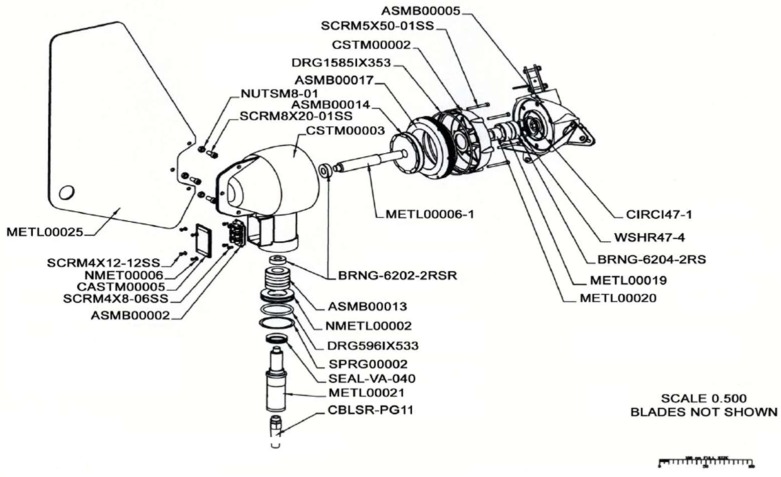
Wind Turbine.

The system is equipped with two marine current turbines (Figure 11) and a wind turbine (Figure 12) with the following characteristics.

Marine current turbines:

- Model: Underwater 100.
- Characteristics: 100 W (24 V). One clockwise & one counter clockwise propeller have been chosen in order to avoid undesirable moments.
- Manufacturer: Ampair [34].

Wind Turbine:

- Model: Ampair 600.
- Characteristics: 600 W, rotor diameter 1.7 m. Automatic pitch control with high winds.
- Power: 600 W (24 V).
- Manufacturer: Ampair.

The storage of the generated energy dedicated to the self-consumption of electronic equipment is carried out in a system of 2 AGM batteries located in a sealed stainless steel box on the platform of the buoy.

**Figure 13:**
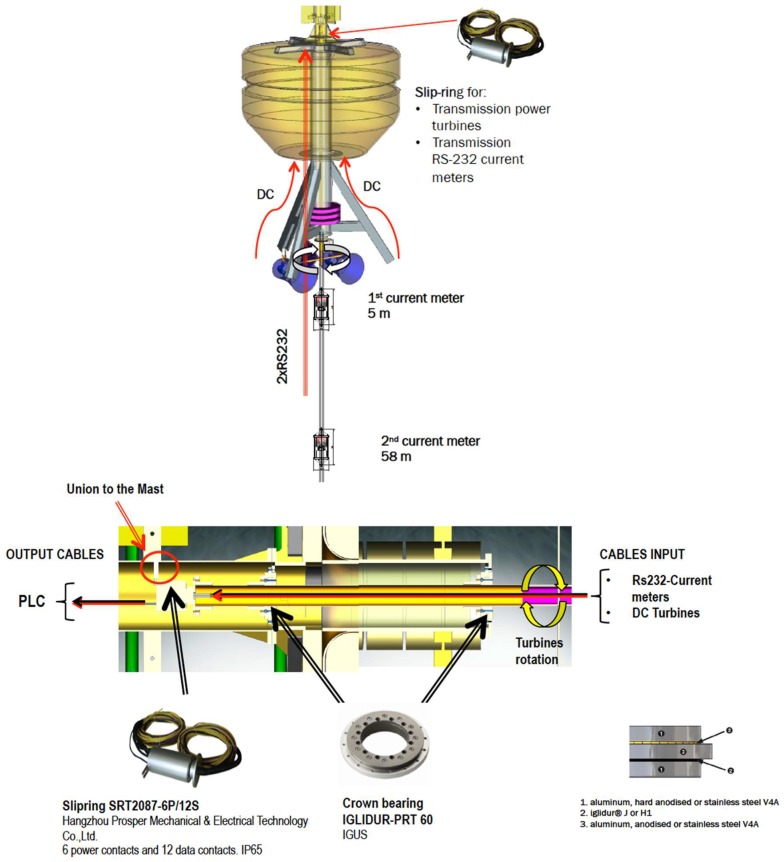
Slip-ring installation.

In the implementation process, a problem of special interest was the transmission of the signal from the current meters sensors and the power generated by the marine current turbines. The rotational movements to be made by the turbines were not compatible with the laying of an uninterrupted wiring (Figure 13). The rotation of the turbines support would cause the tangle-braiding of dangling cables. This problem could be solved using a slip ring [41], a device designed to allow transmission of both power and electrical signals from a stationary to a rotating structure.

#### 2.2.3. Control System and Communications

The control and communications system is a combination of a PLC and a UHF radio. The PLC unit has been programmed to interpret the different sensors protocols (NMEA, RS232, analog), storing and sending the sensor data at regular intervals. A microcontroller-based interface for the adaptation of the digital sensor signals from the wave sensor (manufacturer’s proprietary format SR10) has been implemented. Its main features can be seen in Appendix A.

ACE3600 is a high performance Remote Terminal Unit (RTU) with communication capability. The unit is designed to provide scalability and modularity to optimize the performance of any control system. The unit’s rugged design offers compliance for the requirements of this SCADA system environment. Motorola has developed this innovative RTU to provide a cost effective RTU solution by minimizing the installation and configuration time. 

**Figure 14:**
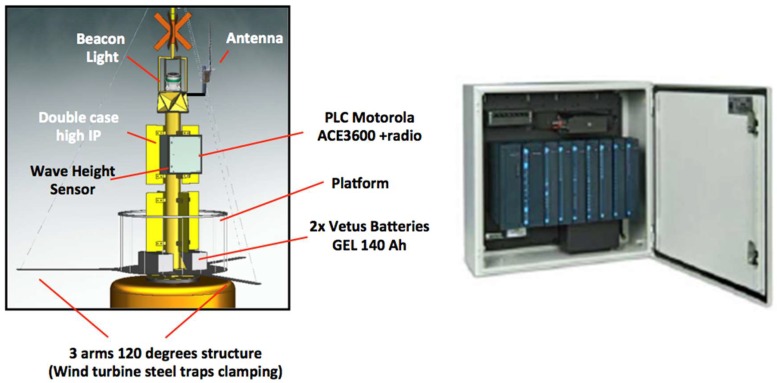
Motorola PLC.

In order to achieve maximum protection and reliability, the RTU was installed inside a double sealed metal enclosure (Figure 14).

**Figure 15:**
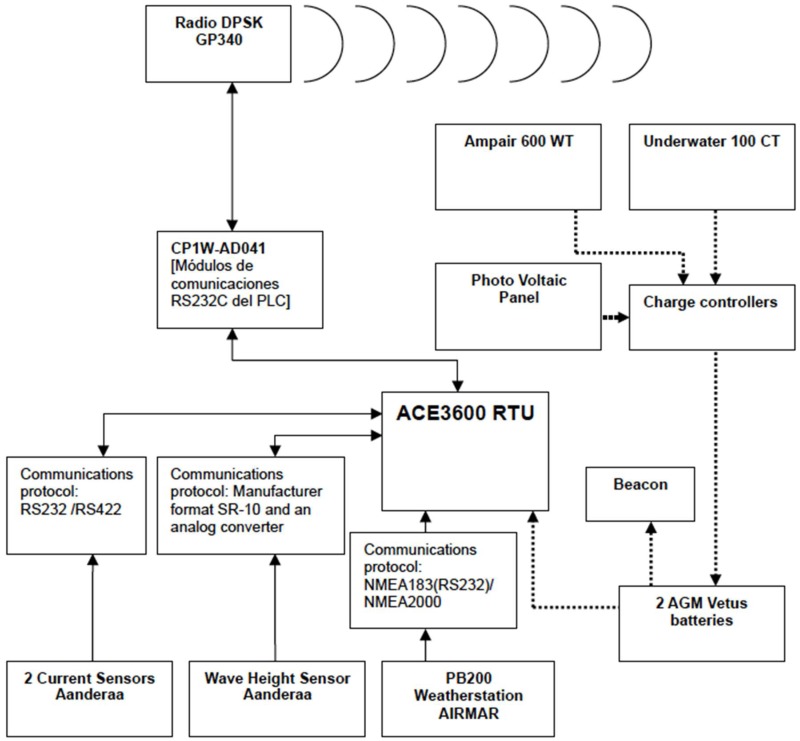
Communication system block diagram.

The ACE3600 RTU facilitates the establishment of a highly sophisticated hybrid data communication network for SCADA that utilizes a variety of radio and/or line communication links. Radio links may include conventional (VHF, UHF, 800 & 900 MHz), analog trunked, digital trunked, and both analog and digital microwave radio technologies. Line links may include point-to-point, multi-drop, Public Service Telephone Network (PSTN) voice/data via dial-up modems, cellular packet data modems, and Local Area Networks (LAN). The implemented communication architecture is shown in Figure 15.

## 3. Results and System Validation

Preliminary studies and tests conducted at Valencia Port demonstrated the need to carry out redesigns to increase the current turbine’s performance. In the selected locations, the wind flows were considerable but sea currents reached average values of 0.5 and a maximum of 0.8 m/s.

**Figure 16:**
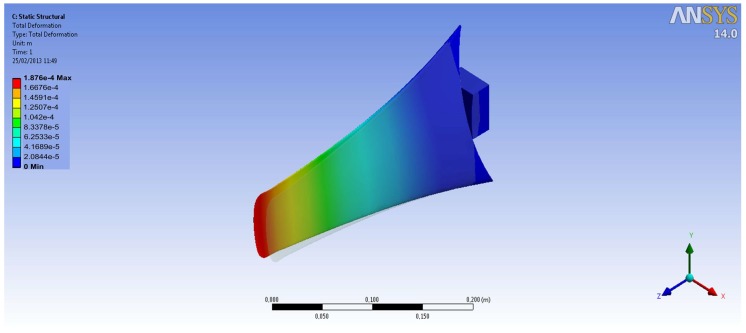
Blades improvements CAD design.

**Figure 17:**
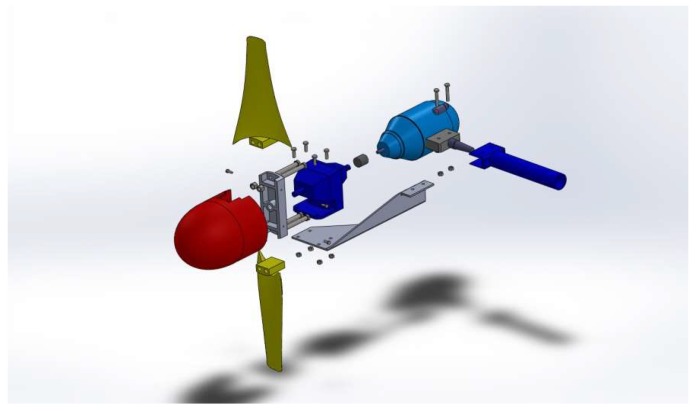
Marine current turbine including a gearbox.

**Figure 18:**
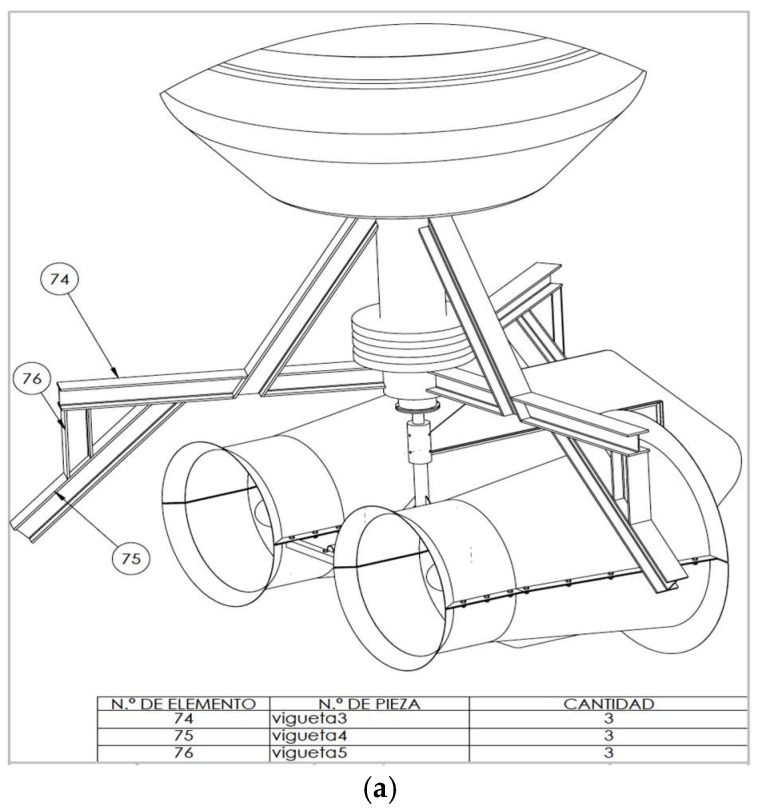

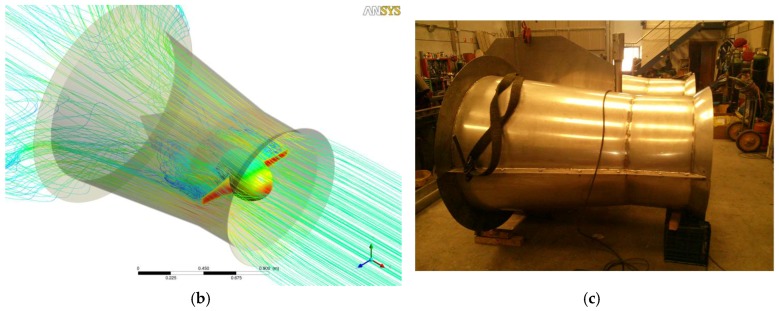
Augmentation channels improvements, CAD design (**a**,**b**) and implementation (**c**).

The improvements objectives were, on the first hand, to redesign the original blades of the marine turbine (Figure 16), since they were commercially designed for direct high-speed seawater drag operation. The introduction of a transmission gearbox was necessary for low value marine currents (Figure 17). Augmentation channels or nozzles were also included to increase the incoming flow at the turbine. Due to modifications made in the blade models and nozzles, it was necessary to make new modifications in the size of the tripod of the anchoring system (Figure 18). 

**Figure 19:**
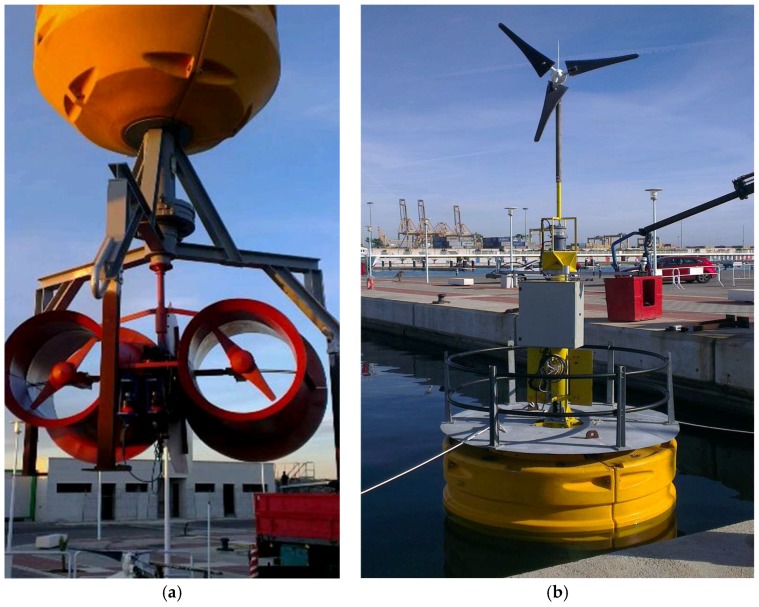
Sensor buoy final design. (**a**) Launching process; (**b**) Moored buoy.

This current speed limitation in the Mediterranean Sea constitutes a motivating factor to intensify research on the increase of the efficiency of MCTs. With this objective, we have developed a procedure of design, simulation, and implementation based on the installation of an augmentation channel in each of the MCTs. We have performed studies based on simulations with different nozzle configurations. Figure 19a shows simulation results considering a current input speed of 201.87 mm/s. An output speed of 513.96 mm/s is obtained. The conic channel surrounding the turbines acts as a current concentrator increasing the incoming flow at the turbine by a factor of 2.71. The validation of the increased flow ratio was done though the FEM module available in the CAD-CAM package Unigraphics NX. In Ref. [31], detailed information on our work can be found.

Figure 19 shows the buoy launching process and the buoy moored in the south side of Port of Valencia.

**Figure 20:**
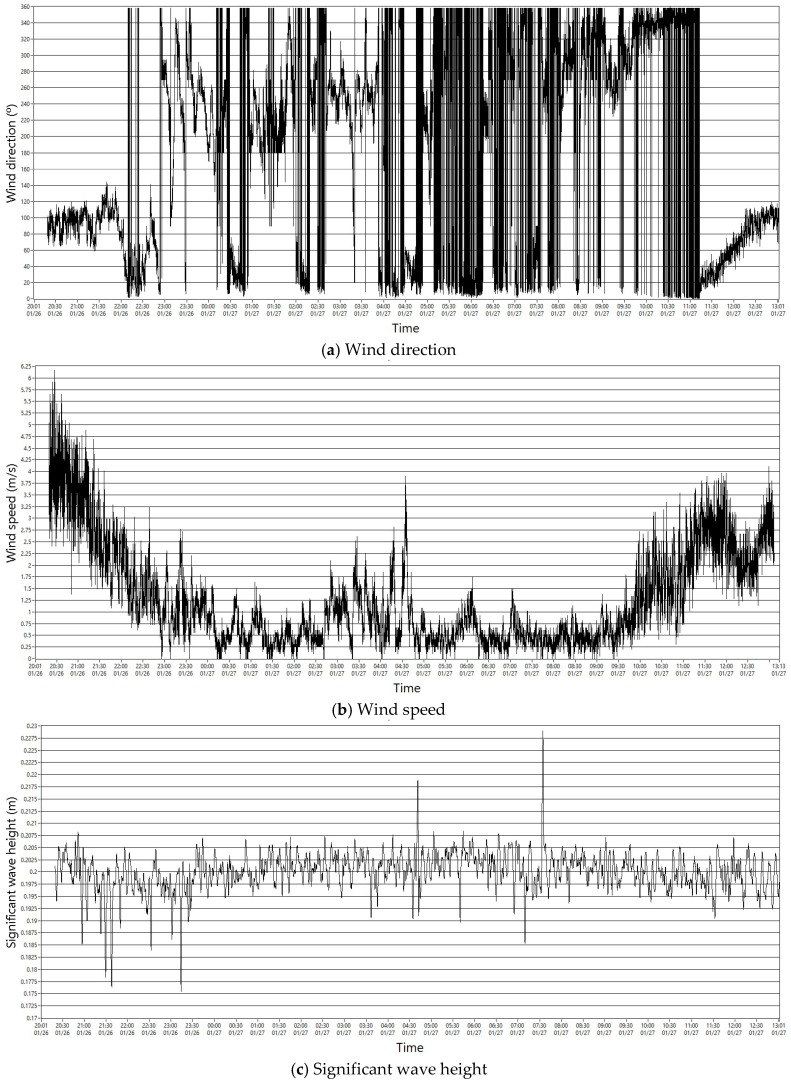

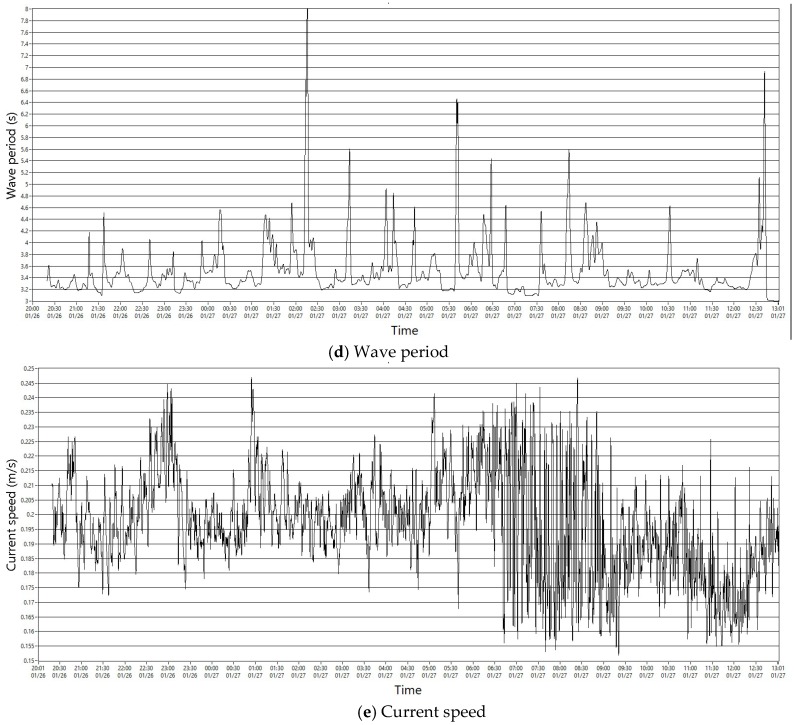
Part of the data collected by the sensor buoy.

In order to check proper communication from all the buoy sensors and actuators, a SCADA system has been used. AIRMAR specifically offers the Weather-Caster free software that can be downloaded from its web page, in order to visualize the data of the PB200 weather-station. Communication can be established via the NMEA183 protocol (RS232) or with NMEA2000 protocol. This software allows the conversion of some of the displays on the screen in time series data. It can store the raw data in .txt files. From a general point of view, all the data were organized through a NMEA2000 network (derived CAN network). Figure 20 shows marine observation data monitored from the buoy sensors.

## 4. Discussion

The measurements that have been obtained and presented in this work should be taken into account fundamentally from the point of view of the proper functioning of the observation system under test, and not from the strict point of view of the specific energy resources existing in open sea areas of the Valencian coast. In this initial phase of the project development, the tests have been carried out in conditions of partial confinement that derive from the initial installation of the buoy in the interior of the southeast area of the Port of Valencia.

From the point of view of monitoring marine energy resources, a floating observation system like the one presented in our work has significant advantages. It allows easy transportation and localization in specific areas subject to energy exploitation monitoring. The Spanish buoys network of Puertos del Estado [42], being a very important tool for the observation of the marine environment, has not been deployed with energy resources monitoring criteria, but rather for the purpose of weather forecasting for navigation aid.

A difference to be highlighted when comparing our system with other existing solutions is that it incorporates a wind generator and marine current turbines, in addition to a solar panel. These additional power sources for the system allow for longer operating autonomy. At the same time, these energy generators can be used to establish local and precise data correlation on the existing resources and their real use in registering long-term data series.

Our system is equipped with a PLC that allows more proactive actions in the design of algorithms for the control of the installed generators, analysis of the system’s stability, and fault diagnosis. The sealed installation box of the PLC and its wiring connections has been developed through the criterion of double redundancy and the storage system of the batteries has been designed in a tight and robust manner so that the spillage of polluting material into the sea is very unlikely. 

Both the access to the buoy and the permanence in it is comparatively friendly, and offers safer conditions for the development of maintenance tasks without the need to transfer them to port.

Compared to smaller solutions, the size and weight of this floating buoy allows the system to experience lower accelerations that may affect the installed electronic devices.

Also, due to its size and characteristics, it allows future expansion in terms of the equipment and the positioning of the sensor devices.

This floating system for parametric observation of marine energy resources has been designed to operate in harsh weather conditions. Therefore, in general, the marine floating observer is intended to be used indistinctly in the Atlantic Ocean or in the Mediterranean Sea. But, in the context of the objectives of this paper, the discussion focuses on the study of energy resources in the Mediterranean Sea environment, which as it is known, has less potential than the Atlantic Ocean. As future work in our project, long period observations tasks have to be made in sites of interest in order to check operation in different sea weather conditions and to get serial data for energy resource analysis.

The existing wave power resources in the Mediterranean Sea, although not being comparable to those existing in the Atlantic zone, are worthy of being considered as candidates for exploitation. Measurements made in our tests and confirmed by other Puertos del Estado buoys, show a significant wave height *Hs* of 1 m and maximum values of 5 m, with different wave period values *Ts* of 4, 5, 6, and 7 s. In the most unfavorable case of waves of *Hs* = 1 m and *Ts* = 4, the power generated would be in the order of *P* = 2 kW/m calculated for deep water conditions. In special situations of strong storms where values of *Hs* = 6 m and *Ts* = 8 are reached, the power generated would be *P* = 144 kW/m. However, economic feasibility studies should include a more precise comparative study that takes into account the optimal performance of different types of wave-based generators. From the general point of view, the best energy resources in the Spanish Mediterranean area are in the western Balearic sea environment. 

The behavioral model of the dynamics of the marine currents is very complex, and until relatively recently (2011), a precise model of the dynamics of the marine currents in the Mediterranean Sea was unavailable. For more detailed knowledge of the general model of currents in the Mediterranean, the study carried out by the NASA/Goddard Space Flight Center can be consulted [43]. In spite of this, the characteristics of local currents as an energy resource require more detailed study.

Preliminary inspections for overlapping marine currents at 60 m depth found no significant differences. This is indicative that it is necessary to observe at greater depths to find the phenomenon of overlapping currents.

Except for particular phenomena, in general, the surface marine currents have greater speed, while the deeper currents provide a higher density. The most significant parameter in (2) is the marine current speed *V*, which is raised to the power of 3. This fact, together with the complications of operation and maintenance of generators located at greater depths, determines its candidacy to be discarded. 

The useful range of operation in marine current turbines is between 1–3 m/s. Under 1 m/s, energy generation is negligible and above 3 m/s is not convenient due to cavitation and overload phenomena [4,44].

Unlike the Strait of Gibraltar and many areas of the Atlantic Ocean in Europe where there are usable currents, the currents observed in the area of the Valencian coast in our work, have a range of 0.2–0.4 m/s, with maximum point values of 0.8 m/s that only allow intermittent operation with prolonged non-operational periods, even with the provision of MCTs TCMs with augmentation channels or nozzles. Therefore, it is convenient to intensify research work to increase the range of operation and performance of MCTs.

Also, as part of future work, there would be room for the exploration of the possible effects of speed increase in surface marine currents in coastal areas of rugged mountain orographic characteristics such as that of the headlands of the southern area of the Valencian state community.

With regard to marine wind energy, the best resources are also found in the western environment of the Balearic Sea, due to the orographic conditions of wind channeling in the Gulf of León due to the straits that form between the Pyrenees, the French Central Massif, and the Alps. In the data provided by the Dragonera buoy [42] it is possible to contrast this fact.

## 5. Conclusions

A multi-sensor floating system to monitor energy resource parameters in the marine environment has been designed. The design of the floating system has been made using software tools for treating the static and hydrodynamic characteristics of offshore marine systems, including anchoring requirements and materials. Sensors of proven quality for monitoring renewable energy marine parameters have been used.

The floating system has been designed to be expandable and reconfigurable. It was also designed for being used as a test bench in the control and diagnosis of marine energy generators. For this reason, a wind turbine and two marine current turbines have been installed. These generators, in a redundant way with the solar panel, guarantee the energy supply of the floating system for long observation periods.

In the next stage of the project, the multi-sensor buoy will be used to monitor local renewable energy resources and will be tested in open sea conditions.

## Figures and Tables

**Table 1:** Catenary segments (×3).

Segment	Type	Length (m)	Section (cm)	kg/m	Weight (kg)
Segment 1	Chain	6	4.5	12	72
Segment 2	Polyester	27	4.3	0.2	5.4
Segment 3	Nylon	27	4.2	0.1	2.7
Segment 4	Chain	6	6.8	29	87
	Total	33		Total	167.1

**Figure A1:**
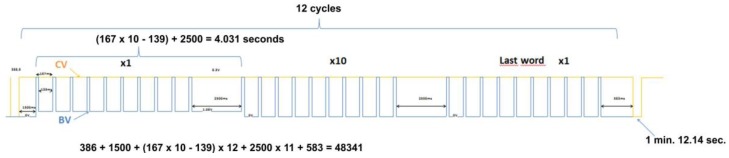
Timeline of the operation of the SR10 signal obtained from measurements with an oscilloscope.

**Figure A2:**
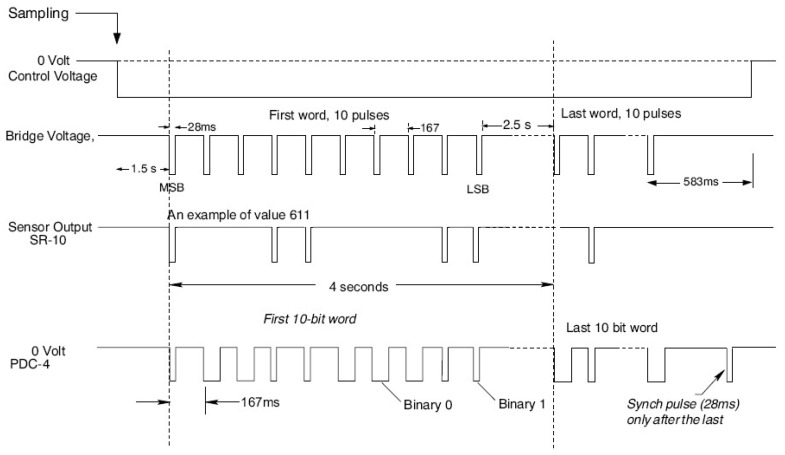
SR10 Timing Diagram.

## Supplementary Materials

Supplementary File 1

## Appendix A

### Appendix A.1. SR10 Signal

In this section the operation of the interface carried out in order to use the wave height sensor using a data format (SR10) from the manufacturer AANDERAA in the MOTOROLA PLC is described.

The SR10 signal is a proprietary format of AANDERAA for the transmission of data to great distances without losses. It works in reverse logic and the mass of the system is positive. It has two control signals CV (Control Voltage) and BV (Bridge Voltage) that activate the sensor. CV works as an “enable”. To activate the sensor, CV must pass from 0 to −6.3 V. After 1500 ms BV must be activated (in blue in the image). BV is a sequence of 12 trains of 10 pulses each where BV passes from OV to −6.3 V with an ON time of 28 ms and an off time of 139 ms. However, the first train of pulses is different from the rest. In the first train of pulses the negative voltage value will be −5 V instead of −6.3 V. The remaining eleven pulse trains will reach the negative value at −6.3 V. The 12 pulse trains are spaced 2500 ms apart after the last rising edge (2500 ms–167 ms if 10 exact pulses are taken into account). After the last train of pulses (last word) (12) there is a wait of 583 ms and CV returns to 0 V until the next shot (CV) is started. Figure A1 shows a recreation of the signal times that were measured with an oscilloscope from the original datalogger.

During the generation of the pulse train and for each falling edge the sensor generates an output (high or low level) that generates a 10-bit word indicating the value of the measurement (wave height or wave period). See Figure A2 for the SR-10 Sensor Output.

### Appendix A.2. PLC Features

Model: PLC/RTU advanced MOTOROLA ACE 3600 (CPU3680).

Communications: 1× RS232/RS485, 2× RS232, 1 × 10/100BaseTX, 2× USB y 1× Modem port for radio.

Input Output Modules:

Module 1: Mixed Module 16 Digital Inputs/16 Digital Outputs (FLN3554)

Module 2: 4 Analog Outputs/8 Analog Inputs ± 5 Volts (FLN3815)

Module 3: 16 Digital Inputs 24 Volts/4 Digital Outputs EE 2 A/4AI ± 20 mA (FLN3815)

Radio system: Radio DPSK GP340.

### Appendix A.3. RTU Features

Power PC based processor provides very high performance.

VX-Works based real-time operating system.

Up to three Ethernet ports.

Up to four serial communication ports.

Up to two radio modem ports.

0,3,5,7 or 8 I/O slot wall mount frames, 19″ rack mount on 8 slot frame.

Expansion frames allow up to 110 I/O modules in a single RTU.

Single and double density I/O modules.

Mixed analog input and output modules.

Hot Swap I/O replacement.

Wide operating temperature range −40 °C to +70 °C.

NEMA 4/IP65 Housing, 40 × 40 cm and 50 × 50 cm.

Two-way/trunking/digital radio models.

AC and DC controlled power supply.

6.5 or 10 Ah Backup battery, smart battery charger.

GPS and NTP for time synchronization.

System building tool for configuration and programming.

Remote firmware and program download.

Compatible with MOSCAD family of RTUs.
